# Is intraoperative neuromonitoring effective in hip and pelvis orthopedic and trauma surgery? A systematic review

**DOI:** 10.1186/s10195-021-00605-8

**Published:** 2021-10-13

**Authors:** Luigi Murena, Giulia Colin, Micol Dussi, Gianluca Canton

**Affiliations:** Orthopaedics and Traumatology Unit, Cattinara Hospital—ASUGI, Strada di Fiume 447, 34149 Trieste, Italy

**Keywords:** Sciatic nerve palsy, IONM, Hip, Pelvis

## Abstract

**Introduction:**

Sciatic nerve injury is an uncommon but potentially devastating complication in hip and pelvis surgery. Intraoperative nerve monitoring (IONM) was applied since the seventies in neurosurgery and spine surgery. Nowadays, IONM has gained popularity in other surgical specialities including orthopaedic and trauma surgery. Aim of this systematic review is to resume the literature evidences about the effectiveness of intraoperative monitoring of sciatic nerve during pelvic and hip surgery.

**Methods:**

Two reviewers (GC and MD) independently identified studies by a systematic search of PubMed and Google Scholar from inception of database to 10 January 2021. Inclusion criteria were: (a) English written papers, (b) use of any type of intraoperative nerve monitoring during traumatic or elective pelvic and hip surgery, (c) comparison of the outcomes between patients who underwent nerve monitoring and patient who underwent standard procedures, (d) all study types including case reports. The present review was conducted in accordance with the 2009 PRISMA statement.

**Results:**

The literature search produced 224 papers from PubMed and 594 from Google Scholar, with a total amount of 818 papers. The two reviewer excluded 683 papers by title or duplicates. Of the 135 remaining, 72 were excluded after reading the abstract, and 31 by reading the full text. Thus, 32 papers were finally included in the review.

**Conclusions:**

The use of IONM during hip and pelvis surgery is debated. The review results are insufficient to support the routine use of IONM in hip and pelvis surgery. The different IONM techniques have peculiar advantages and disadvantages and differences in sensitivity and specificity without clear evidence of superiority for any. Results from different studies and different interventions are often in contrast. However, there is general agreement in recognizing a role for IONM to define the critical maneuvers, positions or pathologies that could lead to sciatic nerve intraoperative damage.

**Level of evidence:**

Level 2.

## Introduction

The sciatic nerve is the largest branch of the sacral plexus, deriving from L4 to S3 spinal nerve roots that reunite to form a single nerve. The nerve enters the grater sciatic foramen and continues down the posterior thigh to the popliteal fossa where it divides in the tibial and common peroneal nerve. The anatomical path of the sciatic nerve increases the risk of injury or palsy during hip and pelvis surgery. Sciatic nerve injury is an uncommon but potentially devastating complication. The rate of injury ranges from 1.5 to 4.7% depending on the type of surgery [1–3]. The most common intraoperative causes are: direct trauma, inappropriate retractor positioning, compression [4] and patient positioning [3]. Risk factors include developmental dysplasia of the hip [5], female gender and revision surgery [6].

### IONM: technical considerations

Intraoperative nerve monitoring (IONM) was applied since the seventies in neurosurgery and spine surgery. Nowadays, IONM has gained popularity in other surgical specialities including orthopaedic and trauma surgery. In the literature, there are several types of IONM described: Motor Evoked Potentials (MEPs), Somatosensory Evoked potentials (SSEP), Electromyography (EMG), Anterior Root Muscle (ARM) response.

MEPs are electric signals recorded from the target group muscles following stimulation of descending motor pathways inside the brain. This type of registration allows to monitor the motor system in its entirety (from cortical motor neuron up to neuro-muscular junction) and to evaluate the limbs separately. Muscle responses are particularly affected by anaesthesia and neuromuscular blockade or pre-existing deficits, and special stimulation techniques and certain anaesthetic regimens are used to optimize MEPs during IONM.

SSEPs monitoring provides a continuous evaluation of the somatosensory system through repetitive stimulation of a peripheral nerve and the recording of multiple responses obtained from the spinal cord and somatosensory cortex. The limits of SSEPs are widely known in literature: no information about motor conduction pathways, up to 20% of false negative [7, 8] or positive [9, 10] and the susceptibility to be influenced by comorbidities (compartment syndrome) and leg positioning [11]. This method does not reflect injuries of the motor tract, but it is less affected by technical difficulties associated with MEPs monitoring. Therefore, the two techniques are often used in combination and the presence of a neurophysiologist in the room is required [12].

ARM response is a non-invasive technique using adhesive electrodes instead of needles, able to record muscles response with continuous low intensity stimuli. It can be performed with the patient under spinal anaesthesia.

EMG detects electric muscle potential in two forms: spontaneous (sEMG) or triggered (tEMG). While the first one provides real time information, the second one needs a neurophysiologist input. Both may be combined with SSEPs or MEPs to improve specificity of the procedure. Those techniques provide information about the intraoperative status of a nerve in different ways, potentially warning the surgeon when the nerve is at risk [13].

## Materials and methods

Two reviewers (GC and MD) independently identified studies by a systematic search of PubMed and Google Scholar from inception of database to 10 January 2021 using various combination of the terms: “intraoperative sciatic nerve monitoring” and “sciatic nerve palsy” and “hip surgery nerve monitoring”. Aim of this systematic review is to resume the literature evidences about the effectiveness of intraoperative monitoring of sciatic nerve during pelvic and hip surgery. The two reviewers screened the titles and abstracts of the citations identified independently and in duplicate, and acquired the full text of any article that either judged potentially eligible. The references list of all the potentially eligible papers were hand-searched and reviewed for any other title not previously found.

The inclusion criteria for the studies were: (a) English written papers, (b) use of any type of intraoperative nerve monitoring during traumatic or elective pelvic and hip surgery, (c) comparison of the outcomes between patients who underwent nerve monitoring and patient who underwent standard procedures, (d) all study types including case reports.

**Fig. 1:**
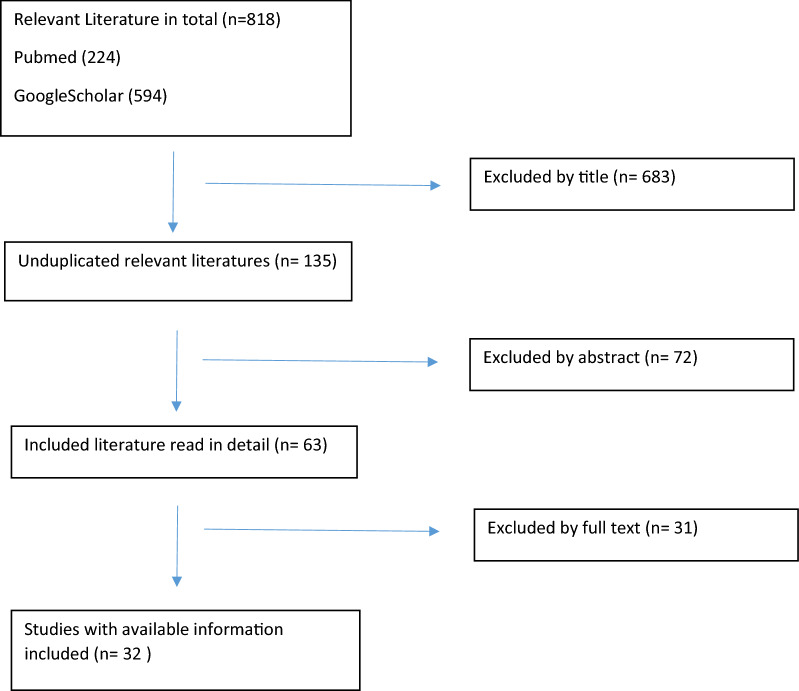
PRISMA flowchart

The exclusion criteria were: (a) language of publication different from English (b) animal research models, (c) book chapters. The present review was conducted in accordance with the 2009 Preferred Reporting Items for Systematic Review and Meta—Analysis (PRISMA) statement [14] (Fig. 1).

## Results

The literature search produced 224 papers from PubMed and 594 from Google Scholar, with a total amount of 818 papers. The two reviewer excluded 683 paperss by title or duplicates. Of the 135 remaining, 72 were excluded after reading the abstract, and 31 by reading the full text. Thus, 32 papers were finally included in the review.

## Discussion

### Periacetabular osteotomy (PAO)

Bernese periacetaboular osteotomy (PAO) is the gold standard in the treatement of acetabular dyspalsia and anomalies of acetabular orientation [15, 16]. Due to the technical difficulties of the procedure and to the steep learning curve, neurovascular complications are possible [17]. In the first report on 75 patients who underwent PAO, Ganz et al. [18] did not refer about any sciatic nerve palsy. Sierra et al. [19] in a 2012 multicenter retrospective study, reported an incidence of sciatic nerve injury up to 15%, decreasing to 2.1% in the experience of highly specialised surgeons. As demonstrated in a cadaveric study, during PAO the sciatic nerve could be damaged during the first and last stages of the osteotomy, especially in case of penetration of the posterior column for more than 1 cm. Moreover, it could suffer from excessive tension during hip flexion [20]. Three in vivo studies evaluated the multimodal sciatic nerve monitoring during PAO [21–23]*.* Pring et al. studied a population of 127 patients who underwent PAO with the help of intraoperative continuous EMG monitoring. They recorded neurotonic discharges signalling potential damage to the nerve in 26% of patients, with one patient (0.7%) reporting permanent neurologic sequelae. No case of neurologic impairment was reported in the group of patients who did not experience neurotonic discharges at intraoperative EMG. The authors concluded that EMG monitoring is a valid tool to lower the incidence of nerve injury, allowing the surgeon to quickly modify the technique when EMG alteration are identified [22]. Sutter et al. in 2011, confirmed this conclusion. In a retrospective study on 7894 patients who underwent complex hip surgeries, a population of 140 PAO was analyzed, including 18 patients undergoing IOMN. Among all the analysed types of surgery, PAO was associated to the higher number of intraoperatory alerts by neurophysiologists. Accordingly, the surgeon modified the procedure immedialtely and no post operative nerve injuries occurred. On the other hand, one post operative neurological damage became evident in the non-monitored group (0.71%). The authors recommend the application of multimodal techniques, considering both descending motors and ascending sensory pathways as the most efficient strategy to alert the surgeon and prevent nerve damage [23] (Table 1).

**Table 1:** PAO surgery

Study	Patients	Unmonitored	EMG + MEP	SEP + MEP	EMG	Sciatic nerve injury with monitoring	Sciatic nerve injury without monitoring
Sutter	69	/	69			1	
Novais	40	/	/	40		0	
Pring	140					7	
Kalhor	800	800					4
Yang	643	/	/			8	

### Total hip arthroplasty (THA)

Primary total hip artrhroplasty (THA) has a reported incidence of post-operative sciatic nerve palsy ranging between 0.08 and 3.7% of cases [24]. The incidence becomes 10 times higher in displastic hips [25]. Stone et al. were the first in 1985 to report on intraoperative evoked potentials monitoring during THA. Among the fifty monitored patients, 20% demonstreated intraoperative SSEP alterations. None had a postoperative sciatic nerve dysfunction [26]. Different authors reported similar data in the following years [27–29]. Rasmussen et al. analysed a cohort of 290 monitored patients undergoing THA and compared them with a group of 485 unmonitored patients. They found an incidence of sciatic nerve palsy of 2.8% and 2.7% respectively, with a 0.7% rate of false negative intraoperatory evidence that became clinically evident after surgery. The authors came to the conclusion that SSEP was not helpful in preventing nerve damages [29]. On the other hand, interesting findings came out from the analysis of the surgical steps that could endanger the nerve according to SSEP intraoperative alterations. The lateral approach appeared to be safer (10%) than the posterior approach (27%), as well as primary THA appeared safer than revision surgery (4.5 vs 19%). Regardless of the surgical approach, the riskier steps appeared to be lateral retraction of proximal femur and anterior retraction of proximal femur [30]. Necessarian et al. reported on 35 patients who underwent revision or reoperation for total hip arthroplasty while monitoring SSEPs. Of the patients who exhibited intraoperative potential changes (32% of cases), due to retractors positioning and limb positioning, none experienced postoperative sciatic nerve compromise. Conversely, two of the 35 unmonitored patient developed inclomplete neurologic palsy. One of them was lost at follow-up, the other recovered completely [31]. MEPs, in combination with EMG, have also been used to study and identify which intraoperative factors may lead to sciatic nerve damage. Twenty-seven patient who underwent revision THA were studied. Electrical changes occurred in 16 cases, most commonly during posterior retractors placement for acetabular exposure using the posterior approach. None of them had post operative nerve impairment. Likely, no nerve palsies were seen in the control group of 113 unmonitored patients. According to the results obtained, the authors could not recommend the use of IONM with combined MEPs and EMG in routine clincal practice, whereas they recognized it was an helpful tool to indicate risk factors for nerve injury [32]. As an alternative to SSEP or MEP, spontaneous electromyography (sEMG) has been introduced in clinical practice. The main advantage is that sEMG records real time muscle activity as a measure of nerve impulses, while SSEP records an average impulse over a specific time interval. Therefore, sEMG has the potential to warn the surgeon in time to prevent nerve injury, as showed by Sutherland et al. [33]. Spontaneous EMG has been proposed also with near-nerve action potentials (NAPs) in a retrospective/prospective study of sixty-three revision total hip arthroplasties. NAPs were intended to be better than SSEPs as they cannot be affected by anesthesia or spinal/epidural blocks. The surgeon was alerted by the neurophysiologist and by audible signals. Fourteen alarms were given to the surgeon during surgical exposure, retraction, lenghtening and wound closure [34]. Sutter et al. [23] confirm that multimodal IONM during THA surgery, both primary or revision, helps to prevent a possible palsy. Of the 44 patients treated, 11 alerts were registered: in 10 cases the alert was resolved after repositiong the leg or shortening the stem, while in 1 case post operative palsy occured despite the corrective manuevers. In revision THA surgery, there seems to be a significant correlation between nerve palsy and post-operatory leg lenghtnening. Both Johanson et al. [35] and Kennedy et al. [36] agreed that restoration of limb length could cause neurological dysfunction. The safe increase of leg length has been reported to be 6–43 mm, in different studies. Recentely, IONM was used to set the critical limit of nerve lenghtnening. There was a direct correlation with anthropometric mesurements as height, weight, distance between the anterior superior iliac spine (ASIS) and the medial malleolus (MM) and the total femoral length. Nerve lenghtening of 5% relative to femoral length and 2.6% relative to ASIS-MM distance was found to be critical [37]. As previously stated, IONM is usually performed in patients undergoing general anesthesia. In the present review there was only 1 study applying IONM to patients in spinal anesthesia. This was possible due to the intrinsic characteristics of ARM. The authors demonstrated the technique to be easy to apply and to give congruent results between intraoperative alarms and postoperative correlations. A total of 20 patients were monitored: none showed alterated signals and there was no post operatory neurological deficit [38]. The literature offers two alternatives to the already described IONM techniques in THA setting. The checkpoint stimulator and the wake up test. The first has been proposed by Shemesh et al. [39] to detect the potential danger of leg lenghtening. It is a single use, handheld, sterile device intended to offer electrical stimulation to identify nerves and testing it, analysing nerve conduction in a very specific point of time. The device does not need specific equipement and does not prolong surgery duration and perioperative setup. On the other hand, it does not provide continuous monitoring. In the study of Shemesh et al. 11 hips were monitored with this technique and in 2 cases a change in nerve response was identified and the maneuvre corrected. They had no permanent postoperative injuries with an average increase of 28.5 mm (6–51 mm) in limb length [39]. The wake up test is mainly used in neurosurgery. The test involves temporarily lightening of the anesthetic depth to the point where the patient is able to follow verbal commands. In the single report available regarding THA surgery, twenty patients (22 hips) underwent the test. In 1 case, the patient could not dorsiflex the foot during the wake-up test after limb lenghtening. Because of that, the surgeons immediately shortened the length of limb. Drop foot resolved completely after 8 weeks. This study support the idea that the wake-up test can be used to check nerve injury during hip surgery and it’s cheaper and more versatile than neuromonitoring [40] (Table 2).

**Table 2:** Hip arthroplasty and hip revision surgery data

Study	Patients	Unmonitored	SEP	sEMG	MEP + EMG	sEMG + NAP	ARM + EMG	HnS	Sciatic nerve injury with monitoring	Sciatic nerve injury without monitoring
Stone et al.	50	/	50						0	/
Rasmussen	775	485	290						8	13
Nercessian	60	35	25						0	2
Black	100	/	100						3	
Bruce (unpublished data)	574	574	/							15
Porter	88	42	46						2	1
Kennedy	23	/	23						0	
Sutherland	44	/	/	44					0	
Satcher	140	113	/	/	27				0	0
Brown	63	/	/	/	/	63			0	
Dikmen	20						20		0	
Pereles	52	/	52						0	
Shemesh	11	/						11	0	
Kong	91	56			35				0	6

### Hip arthroscopy

Nerve injuries in hip arthroscopy surgery are mostly related to prolonged or excessive traction, tipically causing pudendal or sciatic nerve palsy, or direct trauma during portal placement, with the risk of injury to the lateral femoral cutaneous nerve [41, 42]. Some tips are described in the literature in order to reduce traction-related nerve injuries, especially limiting traction time to less than two hours and traction weight to 22.7 kg or less [41, 43–45]. Overzet et al. [46] retrospectively studied 10 hip arthroscopies, monitored with posterior tibial, peroneal, femoral or saphenous intraoperative SSEPs, transcranial electrical motor evoked potentials (TCeMEP), train of four (TOF), and electromyography (EMG). The authors found that the whole group had changes in IONM during the procedure and significant changes in sensory and motor evoked potentials. However, only one patient developed post-operative sciatic nerve sensitive deficit, which resolved two weeks after surgery. Telleria et al. [47], in a prospective study on 60 non-consecutive patients who underwent elective hip arthroscopy with intraoperative nerve monitoring (SSEPs alone or SSEPs combined with tcMEPs) found that the amount of nerve signals seen with SSEPs and tcMEP monitoring was higher than what was clinically identified postoperatively. In this study, 58% (35 patients) had a nerve event during surgery and 7% (4 patients, all of whom have had an intraoperative nerve event) had a clinical neurological deficit: one patient had ankle dorsiflexion weakness resolved within 1 week, another patient complained of tingling in the dorsum of the foot resolved in a few hours and two patients had sciatic nerve neurapraxia, solved within 3 days. They observed that the greatest risk factor for nerve injuries was the maximum traction weight, not the total traction time. On the other hand, Ochs et al. [48] found that 54% of the 35 patient they monitored with SSEP, had significative waveform changes with a recovery of SSEP signal from 2 to over 15 min from the release of leg traction. For these findings, they conclude that SSEP intraoperative monitoring could be useful during hip arthroscopy to prevent neural damage.

### Acetabular and pelvic fractures fixation

Iatrogenic sciatic nerve injury is one of the major complications in acetabular and pelvic fracture surgery, with reported rates ranging from 1 to 18% of cases [49, 50]. Patients with posterior column or wall displacement, post-traumatic sciatic nerve injury [51] and patients with fractures requiring a posterior or extensile lateral approach [52] are the groups with the higher risk of perioperative sciatic nerve damage. Helfet et al. evaluated a series of 50 patients with acute acetabular displaced fractures treated with open reduction and internal fixation while monitoring nerve function with intraoperative SSEP. In this study, thirteen patients (26%), all with posterior wall or posterior column fracture, had preoperative sciatic/peroneal neurological motor deficit. Fourteen patients (28%) demonstrated significant intraoperative SSEP changes with an increase in latency of  ≥  10% and/or a decrease in amplitude  >  50%: two of them had a minor peroneal palsy with loss of one motor grade, solved in a few days. One patient (2%), with normal intraoperative SSEP during the surgical procedure, experienced a severe iatrogenic sciatic nerve neurapraxia with foot drop and loss of motor function, resolved within 4 months. The authors, in conclusion, found an incidence of 0% of iatrogenic intraoperative long-term neurological deficit in this study and recommended the use of intraoperative SSEP monitoring in acetabular fractures surgery, particularly in fractures with posterior column/wall displacement and preoperative sciatic nerve injury [12]. The same authors analysed, in a retrospective/prospective study, a total of 103 patients with acute, displaced acetabular fractures treated with open reduction and internal fixation with perioperative SSEP monitoring, performed at baseline status on the operative limb compared to non- operative limb and during surgery. In this study, the incidence of post-traumatic nerve injury was 29% (30 patients), of which 41% demonstrated waveform changes in the baseline SSEP and 38% in the intraoperative SSEP. The incidence of postoperative neurological deficit was 5% (5 patients), all in patients with fractures with significant posterior wall/column displacement: 5 isolated peroneal division injuries, no tibial division damages and no complete sciatic nerve injuries. In two of the cases of peroneal division injury, there was a significant intraoperative SSEP waveform change, in the other three cases no significant intraoperative SSEP change occurred, due to the fact that only tibial division monitoring was performed. The authors confirmed that perioperative somatosensory evoked potentials (SSEP) monitoring is an effective method to prevent intraoperative sciatic nerve injury and further nerve injury in patients with post-traumatic nerve damage, with strong recommendation to stimulate and monitor independently each sciatic nerve division (both tibial and peroneal) [51]. Helfet et al. in a retrospective study of 28 patients with 30 (2 bilateral cases) vertically unstable pelvic fractures treated with open reduction and internal fixation, recommended to perform both an accurate preoperative neurological examination and preoperative baseline SSEP monitoring as 50% of the study population resulted to have preoperative ipsilateral neurologic injury to the sciatic nerve/lumbosacral plexus. In addition, they advise the use of SSEP intraoperative monitoring in order to avoid lumbosacral root compromise during surgery [53]. Vrahas et al. [52], in a retrospective/prospective study, evaluated the efficacy of SSEP during acetabular and pelvic fracture surgery in 82 patients who presented with preoperative neurological deficit in 34% of cases (29% of acetabular fractures, 46% of pelvic ring injuries and 14% of combined injuries). In the first 40 cases evaluated, three patients (3.6%) had an iatrogenic sciatic nerve injury (none in the pelvic group patient). In all cases, monitoring was not performed or the surgeon reaction to neurophysiologist warnings was not prompt. Conversely, due to higher accuracy in SSEP monitoring by the neurophysiologist and to quicker response by the surgeon to SSEP alerts, in the last 42 patients no sciatic nerve palsies were found and none of the SSEP changes were associated with sciatic nerve injury. The authors underline the role of the neurophysiologist who has to recognize promptly even slight wave changes in SSEP in order to minimize iatrogenic nerve injuries and to allow the surgeon to remove the wrong stimulus to the nerve as soon as possible. Calder et al. [50] also used SSEP for IONM in a population of 88 patients who underwent open reduction and internal fixation for acetabular fractures. While the authors confirmed SSEP monitoring to be useful for detection of intraoperative changes in the physiology of sciatic nerve, they could not find correlations with post-operative functional deficit. In fact, only 32% of patients with an abnormal postoperative interlimb amplitude ratio (<  0.5) had a functional nerve palsy and 26% of patients with normal function showed electrophysiologic abnormalities. In a prospective study performed by Helfet et al. [54] in 1997, 74 patients with acute displaced acetabular fractures were evaluated with spontaneous EMG and SSEP combined. In the authors’ experience, this technique proved to be superior to SSEP alone in detecting earlier sciatic nerve injury. Porat et al. [55] conducted a study involving 60 patients with acetabular and pelvic fractures treated with open reduction and internal fixation. They used three different neuromonitoring methods during surgery: transcranial electric motor evoked potential (tceMEP), peroneal nerve somatosensory evoked potentials monitoring (SSEP) and electromyographic (EMG) monitoring. In this study, tceMEP, with sensitivity of 100% and specificity of 86%, and SSEP, with sensitivity of 75% and specificity of 94%, proved to be effective and safe methods in predicting nerve injury. Conversely, EMG alone or in association with SSEP is not recommended by the authors in pelvic and acetabular surgery for its low sensitivity (20%) and high false-negative rate (80%). According to Haidukewych et al. [49], in a retrospective nonrandomized study involving 256 consecutive acetabular fractures fixation (140 unmonitored procedures and 112 monitored with SSEP alone or EMG and SSEP combined), the use of intraoperative monitoring did not reduce the rate of iatrogenic sciatic nerve injury. They observed a 2.9% of iatrogenic sciatic nerve palsy in the unmonitored group and 8.9% in the monitored one. In addition, among the monitored patients who had postoperative injuries, 70% had no intraoperative alerts. Similarly, Middlebrooks et al. [56] disagree with the routine use of SSEP and electromyography during acetabular fractures fixation. In a series of 129 acetabular fractures treated by senior surgeons with ORIF through Kocher-Langenbeck, combined Kocher-Langenbeck and iliofemoral or extended iliofemoral approach without any intraoperative monitoring method, they reported an overall incidence of 1% of iatrogenic sciatic nerve injury. They argued that the usual techniques applied to protect sciatic nerve such us flexing the knee, removing fascial adhesions from the sciatic nerve and careful attention to the placement of retractors are sufficient precautions to minimize potential risk of sciatic nerve palsy. Conversely, Baumgartner et al. [57] studied the incidence of iatrogenic sciatic nerve in pelvic and acetabular fractures, performed with and without SSEP monitoring. The incidence of sciatic nerve injury was 24% in the unmonitored group while in the monitored group the incidence was 5%. (*p* value  <  0.09). The authors concluded that SSEP monitoring can be helpful to reduce iatrogenic sciatic injury rate especially during the learning curve, when nerve injury rates are prone to be higher. All the results of the studies concerning the use of IONM in acetabular and pelvic fracture surgery are summarized in Table 3.

**Table 3:** Acetabular and pelvic fractures surgery

Study	Patients	SSEP	EMG	SSEP + EMG	tceMEP	No monitoring	Sciatic nerve injury with monitoring	Sciatic nerve injury without monitoring
Helfet et al.	74	24	/	50		/	1	/
Helfet et al.	50	50	/	/		/	1	/
Porat et al.	60	36	49		49	11	5	/
Haidukewych et al.	256	112	/	112	/	140	10	4
Baumgaertner et al.	41	20	/	/	/	21	1	5
Helfet et al.	28	28	/	/	/	/	0	/
Helfet et al.	103	98	/	/	/	/	5	/
Calder et al.	88	88	/	/	/	/	2	/
Vrahas et al.	82	82	/	/	/	/	3	/
Middlebrooks et al.	104	/	/	/	/	104	/	1

### IONM techniques compared

Most IONM techniques have been used in all the different surgical interventions considered, alone or combined, making efficacy comparison difficult. Moreover, the presence of the neurophysiologist for some of the described techniques might create an adjunctive bias for results interpretation. However, based on the results of the present review, some resumptive considerations might be given.

Intraoperative EMG was the most widely used, with experience in PAO, THA, Hip arthroscopy and acetabular and pelvic fractures. In most literature reports, authors considered intraoperative EMG useful for the prevention of nerve injury and preferred if with continuous impulses to the muscles. However, the sensitivity and sensibility increased when EMG was used in combination with one or more of the other techniques described, in particular with SSEP.

SSEP and MEP (both transcranial or muscular) are often used in combination to increase sensitivity and specificity in all the surgeries considered. However, results differed significantly with the type of intervention considered. In PAO and hip arthroscopy SSEP and MEP seemed to help the surgeon to prevent nerve damage, while in THA surgery studies the provided data lead to the conclusion that SSEP and MEP (alone or combined) are not useful in routine practice. It appears that in most cases the alarms were given for reversible maneuvers (such as retraction positioning) that probably would not have given postoperative impact anyway. On the other hand, it has been helpful in setting the critical limit in leg lengthening where the nerve is in danger of being permanently stretched. Conversely, in pelvic and acetabular fracture fixation SSEP and MEP are strongly suggested by the majority of authors and the role of neurophysiologist is also advised to help recognize the smallest change in the biomechanical signals.

## Conclusions

The use of IONM during hip and pelvis surgery is debated. The review results are insufficient to support the routine use of IONM in hip and pelvis surgery. The different IONM techniques have peculiar advantages and disadvantages and differences in sensitivity and specificity without clear evidence of superiority for any. Results from different studies and different interventions are often in contrast. However, there is general agreement in recognizing a role for IONM to define the critical maneuvers, positions or pathologies that could lead to sciatic nerve intraoperative damage. The use of IONM can be recommended as an adjunct in those circumstances in which the surgeon desires immediate intraoperative information regarding the potential of a neurological injury or in high risk cases, including those requiring prolonged surgical time, in patients with high body mass index, in severe deformity correction and in revision surgery.
